# The impact of out-of pocket payments of households for dental healthcare services on catastrophic healthcare expenditure in Iran

**DOI:** 10.1186/s12889-021-11209-6

**Published:** 2021-07-28

**Authors:** Abraha Woldemichael, Satar Rezaei, Ali Kazemi Karyani, Mohammad Ebrahimi, Shahin Soltani, Abbas Aghaei

**Affiliations:** 1 Department of Health Systems, School of Public Health, College of Health Sciences, Mekelle University, Mekelle, Ethiopia; 2 Research Center for Environmental Determinants of Health, Health Institute, Kermanshah University of Medical Sciences, Kermanshah, Iran; 3 Cancer and Immunology Research Center, Research Center for Health Development, Kurdistan University of Medical Sciences, Sanandaj, Iran

**Keywords:** Catastrophic, Dental healthcare, Household, Iran, Out-of-pocket payments

## Abstract

**Background:**

Dental healthcare is the costliest and single most source of the financial barrier to seeking and use of needed healthcare. Hence, this study aims to analyses impact of out-of-pocket (OOP) payments for dental services on prevalence catastrophic healthcare expenditure (CHE) among Iranian households during 2018.

**Methods:**

We performed a cross-sectional analysis to determine the prevalence rate of CHE due to use of dental healthcare services among 38,858 Iranian households using the 2018 Household Income and Expenditure Survey (HIES) survey data of Iran. The WHO approach was used to determine the CHE due to use of dental care services at the 40% of household capacity to pay (CTP). Multiple logistic regression models were used to obtain the odds of facing with CHE among households that paid for any dental healthcare services over the last month while adjusting for covariates included in the model. These findings were reported for urban, rural areas and also for low, middle and high human development index HDI across provinces.

**Results:**

The study indicated that the prevalence of CHE among households that used and did not used dental services over the last month was 16.5% (95% CI: 14.9 to 18.3) and 4.3% (95% CI: 4.1 to 4.6), respectively. The adjusted odds ratio (AOR) for the covariates revealed that the prevalence of CHE for the overall households that used dental healthcare service was 6.2 times (95% CI: 5.4 to 7.1) than those that did not use dental healthcare services. The urban households that used dental healthcare had 7.8 times (95%CI: 6.4–9.4) while the rural ones had 4.7 times (95% CI: 3.7–5.7) higher odds of facing CHE than the corresponding households that did not use dental healthcare services.

**Conclusions:**

The study indicates that out-of-pocket costs for dental care services impose a substantial financial burden on household’s budgets at the national and subnational levels. Alternative health care financing strategies and policies targeted to the reduction in CHE in general and CHE due to dental services in particular are urgently required in low and middle income countries such as Iran.

**Supplementary Information:**

The online version contains supplementary material available at 10.1186/s12889-021-11209-6.

## Introduction

Oral health is a crucial component of population health and contributes to the improvement in people’s well-being. Fair access to dental healthcare services promotes cognitive health and potentially reduces healthcare expenditures. However, realizing affordable, accessible, and optimal use of oral healthcare remained public health as well as an economic challenge in many countries [1–5]. The within and between country disparity in dental healthcare is a common issue [6] and may attribute to the limited dental benefit coverage and inequalities among households in the coverage [7]. The estimated global overall cost (direct and indirect) of dental diseases during 2015 was increased by about a 23.2% when compared with the 2010 estimate, and the per capita dental expenditures and per capita productivity losses from severe tooth loss due to dental diseases, and untreated dental caries were considerably high [8, 9]. Regardless of people’s disparity in age, income, and type of insurance coverage, dental healthcare remained the costliest and single most source of the financial barrier to seeking and use of needed healthcare [10, 11].

One of the main negative consequences of out of pocket (OOP) payment is incurring households with catastrophic healthcare expenditure (CHE). The CHE is occurred when OOP payment for healthcare exceeded a predefined proportion or threshold of a household’s ability to pay [12, 13]. Several factors including dental status, socio-economic status of individuals, demographic characteristics (household size, residence, age, etc.), general health conditions as well as the gross domestic product per capita (GDP) were among the factors associated with CHE [14, 15]. Use of specific healthcare services such as outpatient and inpatient care, prescription drugs and seeking health care for healer traditional are also related to increased probabilities of experience of households with CHE [16–18]. There is some evidence on association between use of dentalcare services and facing households with CHE. A study conducted by Kim and Yang [17] in South Korea on 90,696 households have showed that prevalence of CHE among user and non-user households of dental services was 24.6 and 7.8%, respectively. In another study in China by Sun et al. [15] founded that the prevalence of CHE for user dental services at the 10 and 20% of household income was 8.1 and 3.2%, respectively. While these figures for non-user dental services were 1.8 and 0.5%, respectively.

Most of dental services in Iranian health system are provided by private sector. A study has been reported that more than 90% of dentists are working in the private sector [19]. In addition, dental services are not covered by governmental health insurance in Iran and nearly 20% of total dental services costs are covered by the public health system [20]. Evidence on prevalence of CHE due to dental services in Iran are rarely documented [21]. Thus, to fill this gap in the existing literature, the current study aimed to measure impact of OOP for dental services on prevalence CHE among Iranian households at the national and subnational levels during 2018. The findings may contribute important input for decision-makers to devising mechanisms for preventing further CHE due to use of dentalcare services in Iran and perhaps in other similar contexts.

## Methods

### Setting

Iran is a lower-middle-income country. According to national census 2016, the total population residing in its 31 provinces was about 80 million people (https://www.amar.org.ir/english/Population-and-Housing-Censuses). Iran’s health system has both private and public sectors, where the public sector is organized into primary, secondary, and tertiary healthcare service delivery levels while the private sector into secondary and tertiary levels. Most of the secondary and tertiary healthcare services have health insurance coverage, while the services users should perform coinsurance payments at the point of service delivery [22]. The health transformation plan implemented in 2014 intended to remove the financial barriers of access to the healthcare services of the citizens [23].

### ﻿study design and data source

This study used data extracted from the 2018 Household Income and Expenditure Survey (HIES) of Iran, which is annually collected by the Statistics Centre of Iran (SCI) (https://www.amar.org.ir/english/Statistics-by-Topic/Household-Expenditure-and-Income#287686-statistical-survey). The HIES is a large cross-sectional survey with household as the unit for analysis. The data on households’ sociodemographic characteristics, healthcare service utilization (i.e., dental care utilization) and income and expenditures over the last month were collected through a face-to-face interview of the households’ heads, using the United Nations designed and approved questionnaire. The questionnaire enables to obtain data on sociodemographic characteristics of heads of the households and other members (age, gender, education, household size, marital status, etc.), housing condition (rooms per capita, type of house ownership, house size per square meter) and durable assets of the households (e.g., car, dishwasher, freezer, microwave, vacuum cleaner), monthly households expenditures (e.g., food, health, transportation, furniture, communication, clothing and education), and income of the households in the month before the survey. The HIES included data for the urban and rural resident households from all the provinces in Iran and the households were selected through multistage cluster sampling technique. The province-level data on HDI was accessed from the Institute for Management Research at Radbud University (https://globaldatalab.org/shdi/shdi/IRN/?levels=1%2B4&interpolation=0&extrapolation=0&nearest_real=0). The final sample size used for the analysis comprised of 38,858 households selected.

### Variables

The outcome variable in our study was the CHE for use of dental healthcare services, a binary variable with a value equal to one if the household incurred CHE due to use of dental healthcare services in the last month, otherwise zero. Gender, age, and, education status of the household head, household’s health insurance coverage, wealth index (a proxy for the socioeconomic status), residence (urban/rural), use of dental healthcare services and the human development index [11] of the provinces (scored as low, medium and high) were the potential explanatory variables of the prevalence of CHE due to dental healthcare service.

### Analysis and interpretation

The use of threshold for determining catastrophic expenditures is not only arbitrary but also the estimates may vary based on the approach used [12, 24]. This study used the World Health Organization (WHO) recommended threshold level of 40% and considered CHE if the spending in the last month was greater than or equal to 40% of the household’s capacity to pay [18, 25, 26] because the partial normative food-spending, the normative spending on food, housing and utilities, and the actual food-spending methods commonly uses this threshold level [12]. The analysis involved the following steps were used to calculate the CHE due to dental healthcare services [26]:

First, we determined the household’s CTP by reducing the expenditure for food (as subsistence spending) from the monthly household expenditure. That is, the household’s CTP is non-food expenditure, and we adjusted the value for household size using the following formula:

$$ {\mathrm{Eqhsize}}_{\mathrm{h}}=\mathrm{size}\ {\mathrm{of}\ \mathrm{household}}^{\upbeta} $$

Where Eqhsize_h_ is equalized household’s size and β is the 0.56.

Second, we calculated the poverty line (PL) based on the household’s share of expenditures for food out of the total household expenditures using the following formulas:

$$ {\mathrm{Eqfood}}_{\mathrm{h}}=\frac{{\mathrm{foodexp}}_{\mathrm{h}}}{{\mathrm{Eqhsize}}_{\mathrm{h}}} $$

$$ \mathrm{PL}=\frac{\sum {\mathrm{w}}_{\mathrm{h}}\ast {\mathrm{Eqfood}}_{\mathrm{h}}}{\sum {\mathrm{w}}_{\mathrm{h}}};\mathrm{food}45<{\mathrm{foodexp}}_{\mathrm{h}}<\mathrm{food}55. $$

Where Eqfood_*h*_ the share of is spending for food out of the total household expenditures; *w*_*h*_ is the sampling weight of the households; food_h_ is the household’s food expenditure; food45 – food55 is the mean of households’ food expenditure which ranged from 45 to 55%.

Third, we calculated the subsistence expenditure of the households using the following mathematical equation:

$$ \mathrm{subsistence}\ \mathrm{expenditure}\ \mathrm{of}\ \mathrm{household}=\mathrm{PL}\ast {\mathrm{Eqhsize}}_{\mathrm{h}} $$

Furthermore, when the household’s subsistence expenditure was greater than the expenditure for food, we determined the CTP of household as follows:

$$ {\mathrm{CTP}}_{\mathrm{h}}={\mathrm{total}\ \mathrm{expenditure}}_{\mathrm{h}}-{\mathrm{food}\ \mathrm{expenditure}}_{\mathrm{h}} $$

However, when household’s food expenditure was equal to or greater than the subsistence expenditure, we calculated the CTP as follow:

$$ {\mathrm{CTP}}_{\mathrm{h}}={\mathrm{total}\ \mathrm{expenditure}}_{\mathrm{h}}-{\mathrm{subsistence}\ \mathrm{expenditure}}_{\mathrm{h}} $$

Fourth, the CHE was mathematically determined as follow:

$$ \mathrm{houshehold}\ \mathrm{facing}\ \mathrm{CHE}=\frac{{\mathrm{OOPH}}_{\mathrm{h}}}{{\mathrm{CTP}}_{\mathrm{h}}}\ge 0.4 $$

Where OOPH_h_ is the out-of-pocket payment for healthcare services.

As CHE is a binary variable, we used the multiple logistic regression models to obtain the odds of facing with CHE among households that paid for any dental healthcare services over the last month while adjusting for covariates included in the model. These findings were reported for urban, rural areas and also for low, middle and high HDI across provinces. The findings were considered statistically significant at the *P*-value of less than 0.05 and summarized those variables which were statistically significant.

## Results

Characteristics of households included in the analysis, by dental healthcare service use one month prior to the survey and catastrophic health expenditure during 2018 in Iran are reported in Table 1. The mean age was 50.4 years with a standard deviation (±SD) of 15.6 years. The average monthly household expenditures, healthcare expenditures, and out of pocket payments for dental healthcare were 25.8 ± SD 29.9, 2.5 ± SD 6.1, and 0.2 ± SD 0.4 million Iranian Rials (IRR), respectively. On average, the proportion of households that faced CHE during 2018 in Iran was 4.9% (95% confidence interval (CI): 4.7–5.2) and the overall dental healthcare service utilization was 4.7% (95% CI: 5.04–5.49). Female-headed households (7.2, 95%CI: 6.5–7.9), and rural resident households (5.9, 95% CI: 5.6–6.3) respectively had a higher prevalence of CHE than their corresponding counterparts. A higher proportion of male-headed households (5.0, 95%CI: 4.8–5.2) than the female-headed households (3.1, 95%CI: 2.6–3.6) utilized the dental healthcare service. Again, the prevalence of CHE decreased as the wealth index of the heads of the households increased. There were wide variations in the utilization of dental healthcare service and the prevalence of CHE among households across the provinces in Iran (Appendix 1). The households living in the provinces with medium HDI (5.7, 95%CI: 5.3 to 6.1) than in those residing in provinces with the lowest (3.5, 95%CI: 3.2 to 9.7) and the highest (5.2, 95%CI: 4.8–5.6) HDI utilized dental healthcare.

**Table 1:** Characteristics of households included in the analysis, by dental healthcare service use one month prior to the survey and catastrophic health expenditure during 2018 in Iran

Variables	Households n (%)	Proportion of households (95% CI)	
		Used dental care	Incurred CHE
Demographic variables			
*Sex of household head*			
Male	33,751 (86.9)	5.0 (4.8–5.2)	4.6 (4.3–4.8)
Female	5107 (13.1)	3.1 (2.6–3.6)	7.2 (6.5–7.9)
*Age of household head*			
15–45	16,981 (43.7)	5.3 (5.0–5.7)	3.3 (3.1–3.7)
46–65	14,730 (37.9)	5.2 (4.8–5.5)	4.5 (4.2–4.9)
≥ 66-year-old	7147 (18.4)	2.4 (2.1–2.8)	9.4 (8.7–10.1)
Household had ≤5-year-old child			
Yes	9601 (24.7)	4.9 (4.5–5.4)	3.4 (3.1–3.8)
No	29,257 (75.3)	4.7 (4.4–4.9)	5.4 (5.2–5.7)
Household had ≥66-year-old member			
Yes	8044 (20.7)	2.8 (2.5–3.2)	9.1 (8.4–9.7)
No	30,814 (79.3)	5.2 (5.0–5.5)	3.8 (3.6–4.1)
Socioeconomic variables			
*Educational status of household head*			
Illiterate	9335 (24.0)	2.1 (1.8–2.4)	8.3 (7.7–8.8)
Literate	29,523 (76.0)	5.6 (5.3–5.8)	3.8 (3.6–4.1)
*Wealth index of households*			
Poorest	7730 (19.9)	1.9 (1.7–2.3)	6.7 (6.2–7.3)
Poor	7775 (20.0)	3.3 (2.9–3.7)	6.3 (5.8–6.9)
Middle	7779 (20.0)	4.1 (3.7–4.6)	4.0 (3.6–4.5)
Rich	7785 (20.0)	6.1 (5.6–6.7)	4.2 (3.7–4.6)
Richest	7789 (20.1)	8.1 (7.5–8.7)	3.4 (2.9–3.8)
*Insurance coverage*			
Yes	34,369 (88.4)	4.9 (4.7–5.1)	5.0 (4.9–5.3)
No	4489 (11.6)	3.4 (2.9–3.9)	4.0 (3.5–4.6)
Ecological variables			
*Geographical location of household*			
Urban	20,312 (52.3)	5.7 (5.4–6.1)	4.0 (3.7–4.3)
Rural	18,546 (47.7)	3.6 (3.3–3.9)	5.9 (5.6–6.3)
HDI^*^of province			
Low	14,050 (36.2)	3.5 (3.2–9.7)	4.4 (4.1–4.7)
Medium	12,716 (32.7)	5.7 (5.3–6.1)	5.8 (5.5–6.2)
High	12,092 (31.1)	5.2 (4.8–5.6)	4.6 (4.3–5.1)

**Note**: HDI is human development index; CI is the confidence interval; CHE is the catastrophic healthcare expenditure

Table 2 summarizes the extent of the catastrophic expenditures for households that used and did not use dental healthcare services. While the prevalence of CHE among households with dental care utilization over the last month was 16.5% (95% CI: 14.9 to 18.3), this figure for households without any dental care utilization was 4.3% (95% CI: 4.1 to 4.6). As demonstrated in the table, the rate of CHE for households that used and did not use dental healthcare services in urban area was 16.4% (95% CI: 14.4 to 18.6) and 3.2% (95% CI: 3.0 to 3.5), respectively. The proportion of households faced CHE with and without use of any dental care utilization in rural area was 16.8% (95% CI: 14.2 to 19.8) and 5.6% (95% CI: 5.2 to 5.9), respectively. The illiterate headed-households (19.2, 95%CI: 14.6–24.8) incurred CHE more than twice of those that did not use dental healthcare services.

**Table 2:** Catastrophic healthcare expenditure among households by status of dental care services use during 2018 in Iran

Variables	Percentage of households with CHE (95% CI)	
	Used dental care	Did not use
**Demographic variables**		
*Sex of household head*		
Male	16.1 (14.4–17.9)	4.0 (3.8–4.2)
Female	22.1 (16.2–29.4)	6.7 (6.1–7.4)
*Age of household head (years)*		
15–45	14.9 (12.7–17.4)	2.7 (2.5–2.9)
46–65	17.1 (14.5–19.9)	3.9 (3.5–4.2)
*≥* 66	22.9 (17.3–29.9)	9.1 (8.4–9.7)
*Household had ≤ 5-year old child*		
Yes	15.8 (12.8–19.4)	2.7 (2.4–3.1)
No	16.8 (14.9–18.9)	4.9 (4.6–5.2)
*Household had ≥ 66-year old members*		
Yes	19.2 (14.6–24.8)	8.7 (8.1–9.4)
No	16.2 (14.4–18.1)	3.2 (2.9–3.4)
**Socioeconomic variables**		
*Educational status of household head*		
Illiterate	20.3 (15.2–26.5)	8.0 (7.5–8.6)
Literate	16.1 (14.4–17.9)	3.1 (2.9–3.4)
*Wealth index of households*		
Poorest	20.5 (14.7–27.8)	6.5 (5.9–7.1)
Poor	19.8 (15.3–25.1)	5.8 (5.4–6.4)
Middle	15.2 (11.6–19.5)	3.5 (3.1–4.0)
Rich	16.8 (13.8–20.5)	3.3 (2.9–3.8)
Richest	14.8 (12.2–17.8)	2.3 (2.0–2.7)
*Insurance coverage*		
Yes	16.6 (14.9–18.4)	4.4 (4.2–4.7)
No	16.3 (11.2–23.1)	3.6 (3.1–4.2)
**Ecological variables**		
*Geographical location of household*		
Urban	16.4 (14.4–18.7)	3.2 (3.0–3.5)
Rural	16.8 (14.1–19.8)	5.6 (5.2–5.9)
*HDI of province*		
Low	13.4 (10.6–16.7)	4.1 (3.7–4.4)
Medium	17.2 (14.6–20.1)	5.1 (4.7–5.5)
High	18.3 (15.5–21.5)	3.9 (3.6–4.2)

**Note**: HDI is human development index; CI is the confidence interval; CHE is the catastrophic healthcare expenditure

The regression analysis identified the factors significantly associated with CHE. Both the crude odds ratio (COR) and adjusted odds ratio (AOR) for the covariates revealed statistically significant associations between the prevalence of CHE and the households’ dental healthcare service utilization, residence (urban and rural) as well as the provinces’ HDI status (Table 3). The AOR for the prevalence of CHE for the overall households that used dental healthcare service was 6.2 times (95% CI: 5.4–7.1) than those that did not use dental healthcare services. The urban households that used dental healthcare had 7.8 times (95%CI: 6.4–9.4) while the rural ones had 4.7 times (95% CI: 3.7–5.7) higher odds of facing CHE than the corresponding households that did not use dental healthcare services. The likelihood of incurring CHE also showed a statistically significant increase with the increase in HDI of the provinces.

**Table 3:** Crude and adjusted odds ratio showing the relationship of explanatory variables between paid for catastrophic dental healthcare expenditure of households in Iran

	Crude odds ratio (95% CI)	Adjusted odds ratio (95% CI)
Overall households^a^	4.3 (3.8–4.9)^*^	6.2 (5.4–7.1)^*^
*Urban/rural status*^*b*^		
Urban area	5.8 (4.9–6.9)^*^	7.8 (6.4–9.4)^*^
Rural area	3.4 (2.8–4.2)^*^	4.7 (3.7–5.7)^*^
*Province category based on HDI*^*c*^		
Low	3.6 (2.8–4.8)^*^	5.5 (4.1–7.4)^*^
Medium	3.8 (3.1–4.7)^*^	5.6 (4.5–7.1)^*^
High	5.5 (4.4–6.9)^*^	7.4 (5.9–9.5)^*^

**Note**: ^a^ adjusted for age of household head, gender of head of household, education status of head of household, health insurance coverage, households with under 5 year old children, member of 66 years and above, wealth index of household, residence, and province category based on HDI; ^b^ adjusted for age of head of household, gender of head of household, education status of head of household, health insurance coverage, households with under 5 year old children, member of 66 years and above, wealth index of household and province category based on HD; ^c^ adjusted for age of head of household, gender of head of household, education status of head of household, health insurance coverage, member of 5 years and under, member of 66 years and above, wealth index of household and residing of area. * Significant level < 0.001

## Discussion

The current study indicated that prevalence of CHE was more common among households that used dental healthcare services (16.5%) than among those that did not used any dental care services (4.3%). Similar to the our findings, a study in South Korea found that the prevalence of CHE among households that used and did not used dental services was 24.6 and 7.8%, respectively [17]. The overall prevalence of CHE due to dental healthcare services observed in our study is considerably higher than the findings of many studies in Iran on CHE [18, 21, 25, 27]. Another study showed that OOP expenses for dental services may put a financial significant burden on households [15]. Based on present study results and those aforementioned studies, the prevalence of CHE due to dental healthcare services was much greater; suggesting that dental healthcare utilization had a financial significant burden on household budget in Iran.

The present study showed that health insurance coverage is not provide the financial protection to use of dental care in Iran. The prevalence of CHE among households with and without health insurance that used dental services over the last month was 16.6 and 16.3%, respectively. Sun et al. [15] concluded that there is no association between catastrophic dental healthcare expenditure and health insurance in China. This finding could be explained by this fact that a large proportion of healthcare expenditure are financed by the out-of-pocket payments in Iran. A study indicated that more than 50% of total health costs in Iran are financed by the OOP payments [28]. Another potential factor is that not only most items of dental treatment are not covered by health insurance (less than 20%) but also small fraction of population is covered by dental health insurance [20].

Our empirical analysis revealed the statistically significant positive association between incurring CHE and dental healthcare service utilization. Regardless of the residence of the households, dental healthcare service utilization was associated with an increased likelihood of incurring CHE. The households that used dental care over the last month had nearly fifth increased likelihood of facing the CHE (AOR: 4.3) than the households ones. A study in 41 low- and -middle income countries indicated that that wealthier, urban and larger households and more economically developed countries had higher odds of facing catastrophic dental healthcare expenditure [29]. Our findings revealed a statistically significant increase in the likelihood of incurring CHE due to dental care utilization with an increase in the HDI of the provinces, implying the existence of an “inverse care law” in dental healthcare [30, 31]. Others also reported the statistically significant positive association between the dental healthcare service utilization and the availability of dentists and dental hygienists [32]. However, our findings invite the need for further exploration to identify the real factors that contributed to the increased probability of the occurrence of CHE due to use of dental services among households as the HDI of the provinces increased.

This study for the first time attempted to measure the impact of OOP payments for dental services on prevalence of CHE among the Iranian households. Despite the comprehensiveness of the analysis, our study has some limitations that our study findings should be interpreted with caution. Investigating the main factors associated with the increased chance of incurring CHE among the rural and urban resident households that used dental services as well as the increased occurrence of CHE with an increased HDI of the provinces could have paramount importance for an informed decision in the real context. However, this study has a limitation on uncovering those potential factors. Another limitation is that this study is a cross-sectional design; thus, we are not able establish any causal relationship between prevalence of CHE due to dental care utilization and explanatory potential variables included in the study.

## Conclusion

The findings indicated statistically significant associations of the increased probability of incurring CHE with households’ dental healthcare service utilization. Alternative health care financing strategies and policies targeted to the reduction in CHE in general and CHE due to dental services in particular are urgently required in low and middle income countries such as Iran. However, the success of any polices may depend on pointing out the main contributing factors to the increased chance of incurring CHE due to use of dental care based on the residence of the households and the HDI of the provinces.

## Supplementary Information

**Additional file 1.**

## Data Availability

The data that support the findings of this study are of public domains and available from the websites of the Statistics Centre of Iran (SCI) (https://www.amar.org.ir/english/Iran-Statistical-Yearbook/Statistical-Yearbook-2018-2019) and the Institute for Management Research at Radbud University (https://globaldatalab.org/shdi/shdi/IRN/?levels=1%2B4&interpolation=0&extrapolation=0&nearest_real=0).

## Abbreviations

AOR: Adjusted odds ratio
CHE: Catastrophic healthcare expenditure
COR: Crude odds ratio
GDP: Gross domestic product
HDI: Human development index
HIES: Household Income and Expenditure Survey
OOP: Out-of-pocket payment
SCI: Statistics Centre of Iran
UHC: Universal health coverage
WHO: World Health Organization.
