# Supplementary material for: The impact of out-of pocket payments of households for dental healthcare services on catastrophic healthcare expenditure in Iran
Source: BMC Public Health. 2021 Jul 28;21:1474. doi: 10.1186/s12889-021-11209-6 (PMC8320192; doi:10.1186/s12889-021-11209-6)
Supplement: Supplementary file 1 — Additional file 1. [file 12889_2021_11209_MOESM1_ESM.docx]

| Variables | n (%) | Percentage of households with dental care utilization in the last month (95% confidence interval) | Percentage of households with catastrophic healthcare expenditure (95% confidence interval) |
| --- | --- | --- | --- |
| Tehran | 2019 (5.2) | 5.2 (4.3 to 6.2) | 2.5 (1.9 to 3.2) |
| Gilan | 1315 (3.4) | 4.1 (3.1 to 5.3) | 4.2 (3.2 to 5.4) |
| Mazandaran | 1026 (2.6) | 4.9 (3.8 to 6.4) | 7.3 (5.9 to 9.1) |
| East Azerbaijan | 1279 (3.3) | 9.4 (7.9 to 11.1) | 9.9 (8.4 to 11.6) |
| West Azerbaijan | 1141 (2.9) | 4.9 (3.8 to 6.4) | 2.4 (1.7 to 3.5) |
| Kermanshah | 1373 (3.5) | 8.0 (6.7 to 9.5) | 5.5 (4.4 to 6.8) |
| Khuzestan | 1384 (3.6) | 2.3 (1.6 to 3.2) | 6.9 (5.5 to 8.2) |
| Fars | 1486 (3.8) | 4.2 (3.3 to 5.3) | 6.2 (5.1 to 7.5) |
| Kerman | 1089 (2.8) | 0.4 (0.1 to 1.0) | 2.3 (1.5 to 3.4) |
| Razavi Khorasan | 1608 (4.1) | 6.0 (5.0 to 7.4) | 6.8 (5.6 to 8.1) |
| Esfahan | 1338 (3.4) | 9.1 (7.7 to 10.7) | 6.8 (5.6 to 8.2) |
| Sistan and Baluchestan | 1482 (3.8) | 0.6 (0.3 to 1.1) | 3.8 (2.9 to 4.9) |
| Kurdistan | 821 (2.1) | 5.1 (3.8 to 6.8) | 3.6 (2.6 to 5.2) |
| Hamadan | 1362 (3.5) | 4.6 (3.6 to 5.8) | 3.3 (2.5 to 4.4) |
| Chahar Mahall and Bakhtiari | 1165 (3.0) | 8.5 (7.1 to 10.3) | 4.0 (3.0 to 5.4) |
| Lorestan | 1046 (2.7) | 2.2 (1.5 to 3.3) | 7.9 (6.4 to 9.7) |
| Ilam | 1007 (2.6) | 4.4 (3.2 to 5.8) | 2.7 (1.8 to 3.9) |
| Kohgiluyeh Buyer Ahmad | 1126 (2.9) | 8.8 (7.3 to 10.6) | 2.7 (1.9 to 3.8) |
| Bushehr | 1116 (2.9) | 3.2 (2.3 to 4.4) | 2.6 (1.8 to 3.7) |
| Zanjan | 1119 (2.9) | 3.9 (2.9 to 5.2) | 3.7 (2.7 to 4.9) |
| Semnan | 959 (2.5) | 3.0 (2.1 to 4.3) | 4.4 (3.3 to 6.0) |
| Yazd | 1253 (3.2) | 5.3 (4.1 to 6.6) | 5.4 (4.2 to 6.7) |
| Hormozgan | 1565 (4.0) | 5.0 (4.0 to 6.2) | 4.4 (3.5 to 5.5) |
| Markazi | 1433 (3.7) | 3.7 (2.9 to 4.9) | 5.0 (4.0 to 6.3) |
| Ardebil | 960 (2.5) | 5.2 (3.9 to 6.8) | 5.7 (4.4 to 7.4) |
| Qom | 929 (2.4) | 5.5 (4.2 to 7.1) | 5.9 (4.5 to 7.6) |
| Qazvin | 987 (2.5) | 3.5 (2.5 to 4.9) | 4.8 (3.7 to 6.4) |
| Golestan | 1748 (4.5) | 3.5 (2.8 to 4.5) | 8.7 (7.4 to 10.1) |
| North Khorasan | 1407 (3.6) | 4.2 (3.2 to 5.4) | 6.5 (5.3 to 7.8) |
| South Khorasan | 1356 (3.5) | 1.6 (1.0 to 2.4) | 1.7 (1.2 to 2.6) |
| Alborz | 959 (2.5) | 6.3 (4.9 to 8.1) | 3.6 (2.6 to 5.0) |

Appendix 1: Proportion of households with dental care utilization and faced catastrophic healthcare expenditure across provinces in Iran, 2018
